# Supplementary material for: Hydrogen-induced tunable remanent polarization in a perovskite nickelate
Source: Nat Commun. 2024 Jun 3;15:4717. doi: 10.1038/s41467-024-49213-0 (PMC11148064; doi:10.1038/s41467-024-49213-0)
Supplement: Supplementary file 1 — Supplementary Information [file 41467_2024_49213_MOESM1_ESM.pdf]

Supporting Information

Hydrogen-induced tunable remanent polarization in a perovskite nickelate

Yifan Yuan[1,†,*], Michele Kotiuga[2,†,*], Tae Joon Park[3,†,*], Ranjan Kumar Patel[1], Yuanyuan Ni[4], Arnob Saha[5], Hua Zhou[6], Jerzy T. Sadowski[7], Abdullah Al-Mahboob[7], Haoming Yu[3], Kai Du[8], Minning Zhu[1], Sunbin Deng[3], Ravindra S. Bisht[1], Xiao Lyu[9], Chung-Tse Michael Wu[1], Peide D. Ye[9], Abhronil Sengupta[5], Sang-Wook Cheong[8], Xiaoshan Xu[4], Karin M. Rabe[8], Shriram Ramanathan[1,*]

[1]Department of Electrical & Computer Engineering, Rutgers, The State University of New Jersey, Piscataway, NJ 08854, USA

[2]Theory and Simulation of Materials (THEOS), National Centre for Computational Design and Discovery of Novel Materials (MARVEL), École Polytechnique Fédérale de Lausanne (EPFL), CH-1015 Lausanne, Switzerland

[3]School of Materials Engineering, Purdue University, West Lafayette, IN 47907, USA

[4]Department of Physics and Astronomy, University of Nebraska–Lincoln, Lincoln, NE 68588, USA

[5]School of Electrical Engineering and Computer Science, The Pennsylvania State University, University Park, PA 16802, USA

[6]X-ray Science Division, Advanced Photon Source, Argonne National Laboratory, Lemont, IL 60439, USA

[7]Center for Functional Nanomaterials, Brookhaven National Laboratory, Upton, NY 11973, USA

[8]Department of Physics and Astronomy, Rutgers, The State University of New Jersey, Piscataway, NJ 08854, USA

[9]School of Electrical and Computer Engineering and Birck Nanotechnology Center, Purdue University, West Lafayette, IN 47906, USA

[†]These authors contributed equally

[*]Email: Yifan Yuan, yifan.yuan10@gmail.com Michele Kotiuga, mkotiuga@materialsdesign.com

Tae Joon Park, tjp059@gmail.com Shriram Ramanathan, shriram.ramanathan@rutgers.edu

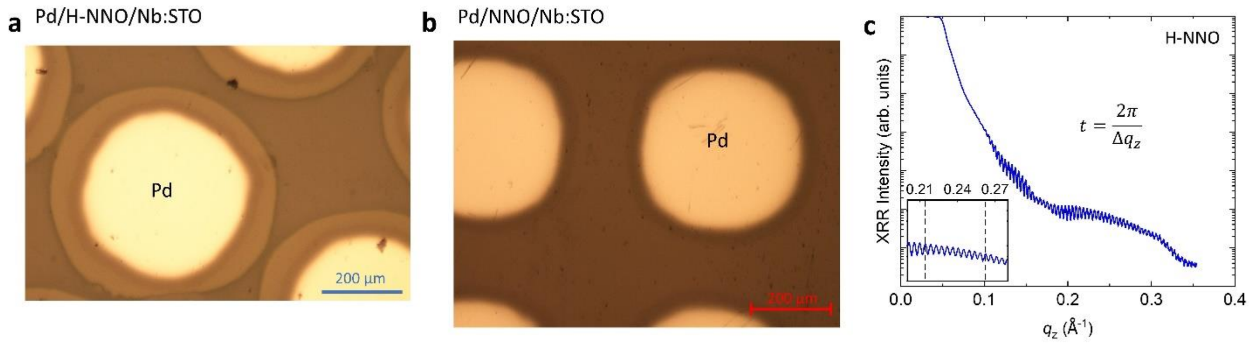

Supplementary Figure S1 | Optical morphology and thickness determination of proton-doped NNO films. The optical image of (a) the H-NNO and (b) the pristine NNO film on the Nb:STO substrates. The bright spots of ~300-µm diameter are Pd electrodes of 50-nm thickness. The circles around Pd electrodes are heavily H-doped regions due to the catalytic spillover. The doping density gradually decays further away from the electrode region. (c) Synchrotron X-ray reflectivity curves for the H-NNO film. The film thickness of 148 nm is estimated with the equation in the figure. The inset is a zoomed-in view over a small thickness range.

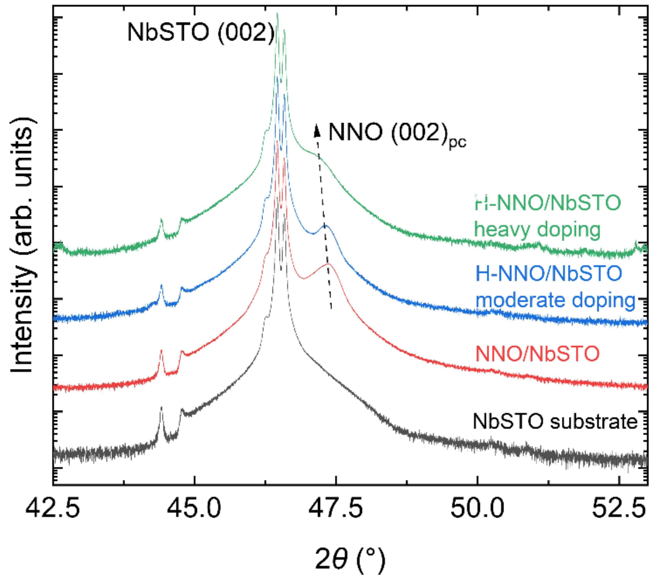

Supplementary Figure S2 | XRD spectra of H-NNO films with different doping levels. θ-2θ Laboratory XRD curves with Cu Kα, β source for Nb:STO substrate, pristine NNO/Nb:STO, moderately doped H-NNO/NbSTO, and heavily doped H-NNO/NbSTO. Moderate doping was performed at 200 °C for 30 minutes under H_2(5%)/Ar(95%) mixture gas. Heavy doping was performed at 250 °C for 8 hours in pure H_2 gas. All film thicknesses are ~100 nm. The dash line marks the change of the $(002)_{pc}$ peak position in the film with the doping levels. The $(002)_{pc}$ peak shifts towards a low diffraction angle and begins to overlap with the substrate peak.

Supplementary Table S1 | Structural parameters of NNO and H-NNO films. Peak positions in RSM Figure 1d,e. in-plane (a_{pc}) and out-of-plane (c_{pc}) lattice constants of pristine NNO and hydrogenated H-NNO films on Nb:STO substrates. The cell parameter $a_{\mathrm{pc,bulk\ NNO}}$ of bulk NNO In the pseudocubic setting[1] is calculated to be 3.807 Å. The lattice constant for Nb:STO substrate with cubic structure is 3.905 Å.

	K	L	a_{pc} (Å)	c_{pc} (Å)
NNO	1.010	2.036	3.866	3.836
H-NNO	1.010	2.011	3.865	3.883

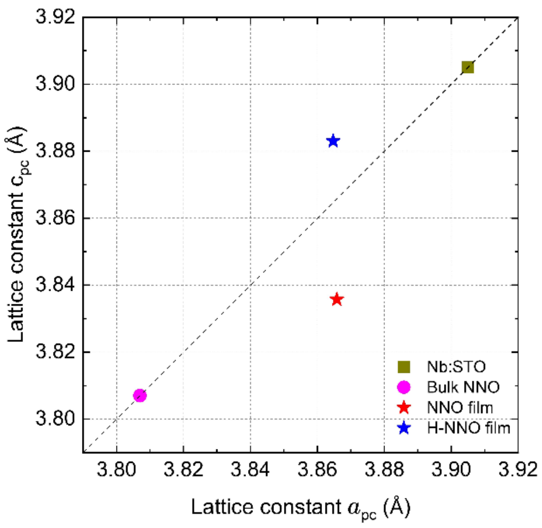

Supplementary Figure S3 | In-plane (a_{pc}) and out-of-plane (c_{pc}) lattice constants of Nb:STO substrate, bulk NNO, pristine NNO films, and H-NNO films.

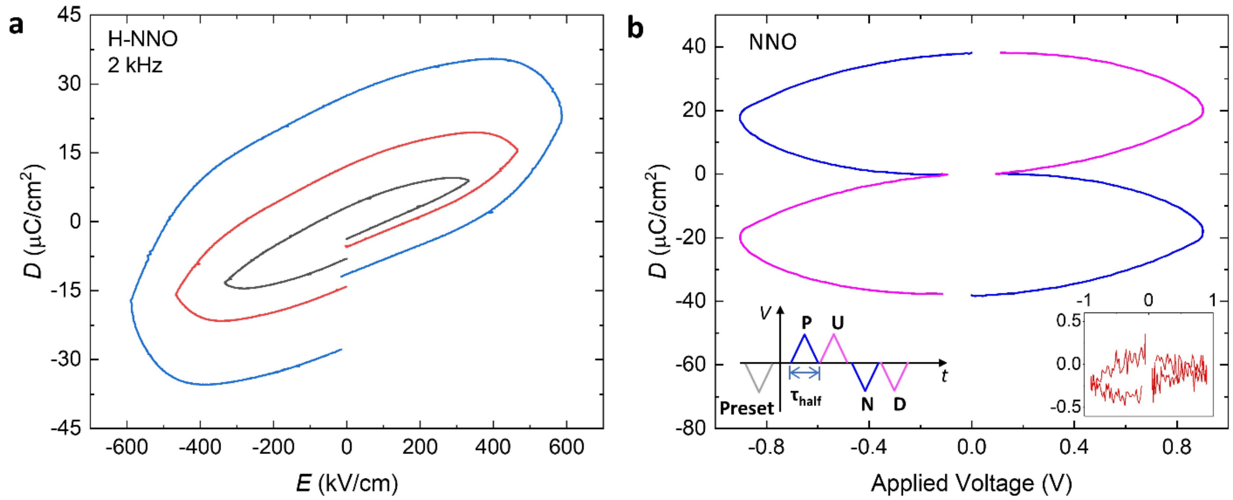

Supplementary Figure S4 | Polarization measurements of H-NNO and NNO films. (a) Electric displacement vs. applied electric field measured at 2 kHz on the H-NNO capacitor. As the electric field increases, the loop becomes more round indicating more contribution due to leakage current. (b) The PUND measurement for the pristine NNO film. The period of half wave τ_{half} is 0.05 ms. Inset shows extracted PUND loop from the measurement.

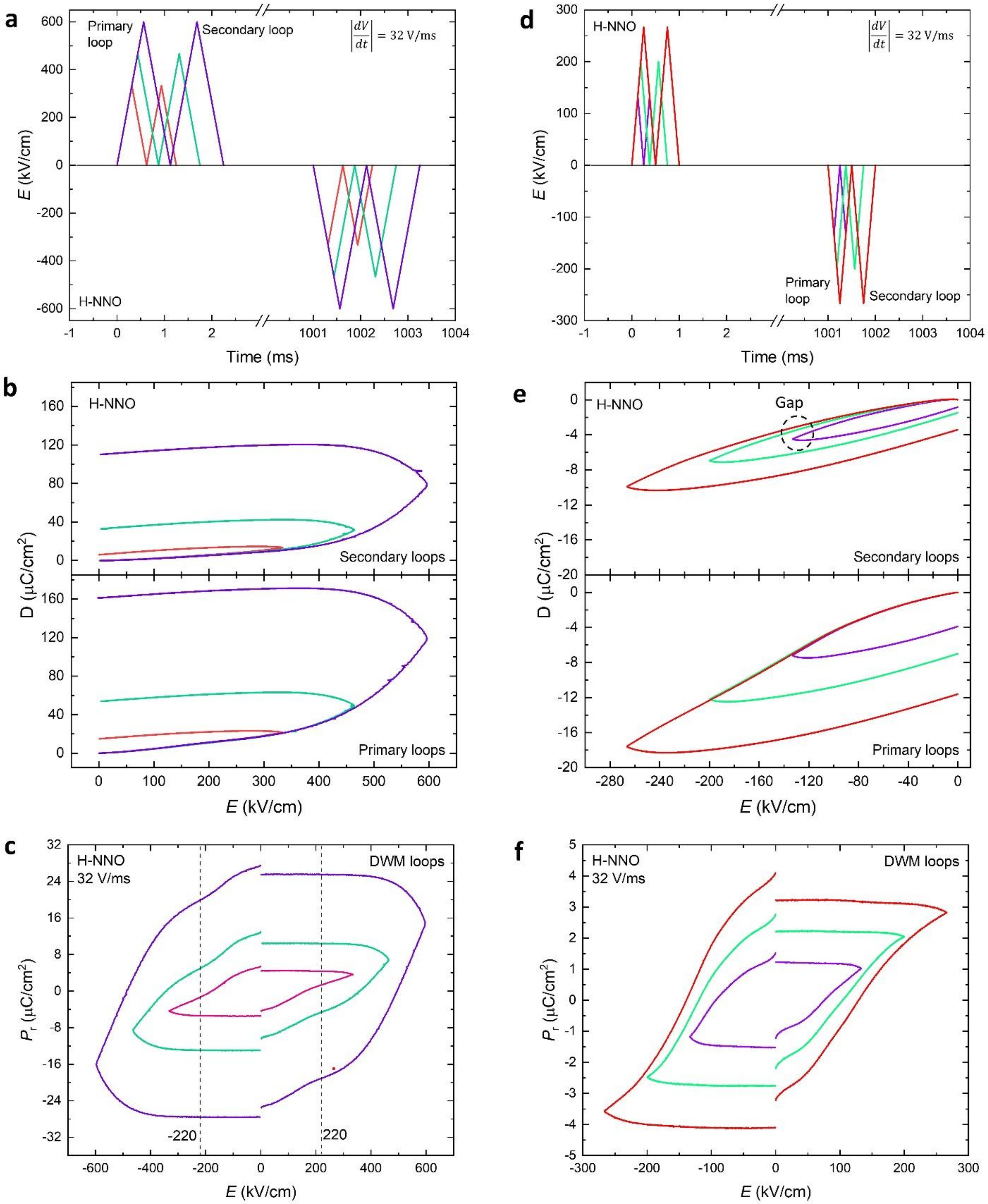

Supplementary Figure S5 | PUND measurements of H-NNO films with different amplitudes of electric fields. (a,d) The applied electric field pulse sequences with the same ramping rate for the PUND measurements at room temperature. Over a series of measurements, the maximum electric field varies from 33 to 631 kV/cm. (b, e) Part of the measured loops. The primary loops overlap well and the secondary loops have differences due to the modulation of remanent polarization. (c, f) the resultant

PUND loops calculated from the difference between the primary loops and secondary loops. It is clear that the PUND loops at high electric field have different shapes compared to ones at low field, implying different mechanisms for the remanent polarization.

Supplementary Note 1 Extracting $P_r(E)$ by modeling the non-switching displacement.

The device is modeled with three components in a parallel circuit: a spontaneous-polarization-only capacitor causing hysteresis, a parasitic capacitor C, and a nonlinear resistor R_v.

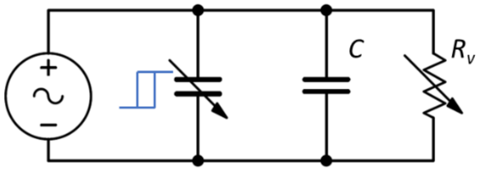

Supplementary Figure S6 | The equivalent circuit for the H-NNO device, consisting of a spontaneous-polarization-only capacitor causing hysteresis, a parasitic capacitor *C*, and a nonlinear resistor R_v.

In the rising ramp of the primary loops (Supplementary Figure **S5**b), the total displacement increases with the increase in electric field and consists of three parts, remanent polarization P_r and non-switching displacement D_{non} caused by dielectric polarization and leakage current, i.e.,

$$D = P_r(V_M) + D_{non}(P_r(V_M), V) \quad (1).$$

As shown in the secondary loops (Supplementary Figure **S5**b,e), D_{non} is affected by P_r which is controlled by maximum voltage V_M in the primary loop, so D_{non} is a function of V_M and V. In the primary loop, when the voltage is increasing, the non-switching displacement is affected simultaneously by $P_r(V_M)$ and V. Because in the rising part, $V_M = V$, so

$$D_{non}(P_r(V_M), V) = D_{non}(V_M = V, V) \quad (2).$$

We experimentally measured the PUND loops with V_M from 1 V to 19 V with the same scan rate. The rising part of primary loops are the same (Supplementary Figure **S5**b,e). The rising ramp of the secondary loops as a function of V_M and V are shown in Supplementary Figure **S7**a,b. These curves $D_{non}(V_M, V)$ are fitted mathematically with MATLAB thin-plate spline interpolation method. Then the envelope of these measured curves is $D_{non}(V_M = V, V)$, acquired from interpolation shown as the light blue curve in Supplementary Figure **S7**a,b. Subtracting $D_{non}(V_M = V, V)$ from the primary loops produces the desired $P_r(V_M)$ relationship (Figure 2f).

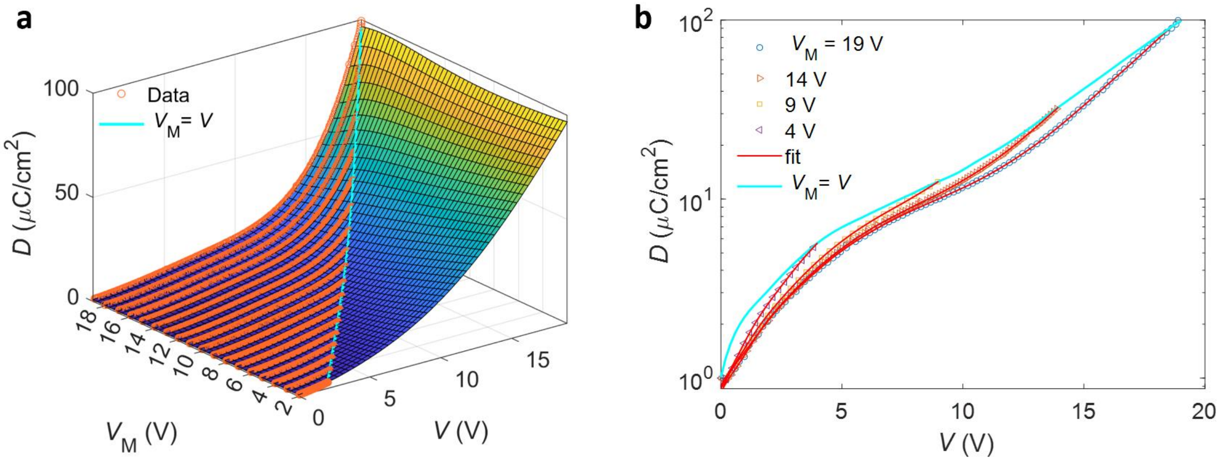

Supplementary Figure S7 | Extracting remanent polarization by fitting non-switching displacement. (a) The surface plot of displacement D_{non} from the secondary loops in Supplementary Figure **S5** as a function of maximum voltage and applied voltage. The meshed surface is from mathematical fitting. The blue curve is the interpolated envelope from the experimental data when V_M=V. (b) Displacement as a function of applied voltage with different maximum voltage. Data are extracted from (a), fitted by the mathmatical interpolation method.

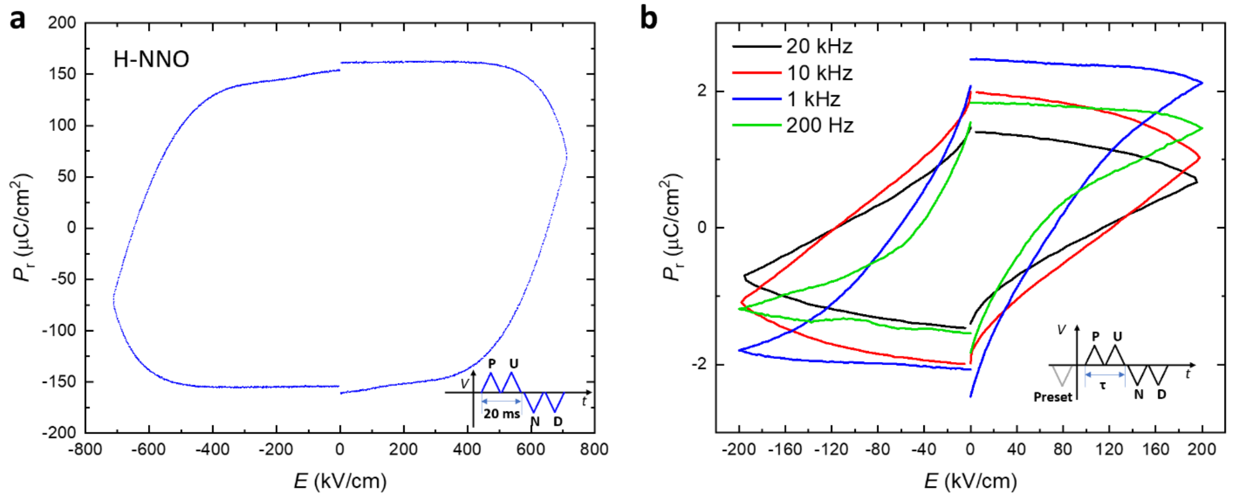

Supplementary Figure S8 | Remanent polarizaiton measurements of H-NNO films. (a) The PUND loop for the H-NNO film at large electric field. (b) PUND loops with different frequencies which corresponds to 1/τ in the inset. The remanent polarization at 1 kHz has the largest value.

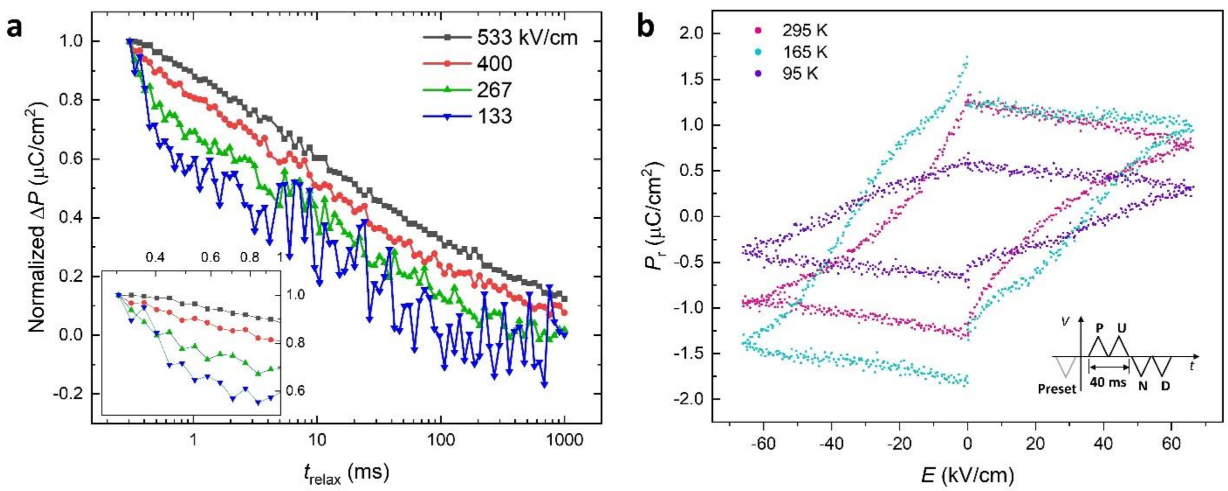

Supplementary Figure S9 | Relaxation of remanent polarization and low-temperature polarization measurements. (a) Normalized ΔP as a function of relaxation time with different maximum electric field. Inset shows the relaxation behavior within 1 ms. Higher initial remanent polarization has slower relaxation behavior. (b) The PUND loops at different temperatures for H-NNO films. P_r reaches maximum value at 165 K.

Supplementary Note 2

An extensive literature review was made on relaxation of remanent polarization in ferroelectrics and electrets, including organic ferroelectric BTA (trialkylbenzene-1,3,5-tricarboxamide)[2] at 75 °C, ultrathin ferroelectric $BaTiO_3$ film of 6.5 nm thickness[3], 170-nm-thick ferroelectric PZT film[4], 140-nm-thick $SrBi_2Ta_2O_9$ film[5], 13-um P(VDF-TrFE) film[6], 11-nm $Hf_xZr_{1-x}O_2$ film[7], 400-nm $BiFeO_3$ film[8], 300-nm $BiFeO_3$ film at 90 K[9], 60-nm-thick $BaTiO_3$ film[10].

Supplementary Figure **S10** shows a summary of remanent polarization vs. relaxation time. The relaxation time is extracted from reports via taking the time interval from initial polarization to 20% of polarization. Most of thick ferroelectric films and single crystals do not have large polarization loss. In ultrathin ferroelectric films, e.g., 6.5-nm-thick BTO, decay of spontaneous polarization is induced by the strong internal depolarization field.

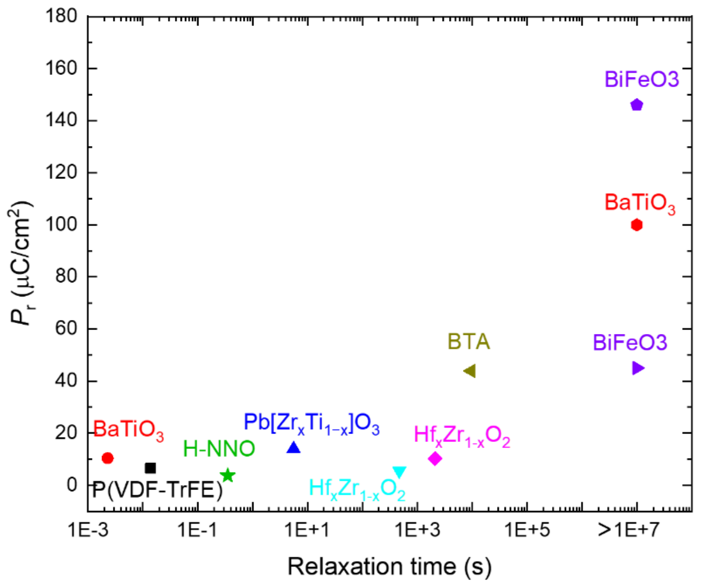

Supplementary Figure S10 | Summary of remanent polarization and relaxation time of different ferroelectric materials. The symbols represent P_r values caused by intrinsic dipole moment. Film thickness details are included in the supplementary note.

Supplementary Table S2 | Fitting parameters for the equation $\Delta P(t_{relax}) = P_0\, exp(-\left(\frac{t_{relax}}{\tau}\right)^{\beta})$. The fitted curves are shown in Fig. 2h.

Electric field (kV/cm)	P_0 (μC/cm^2)	τ (ms)	β
533	164.9	8.48	0.20
400	54.9	3.83	0.21
267	15.5	4.73	0.27
133	4.8	3.22	0.32

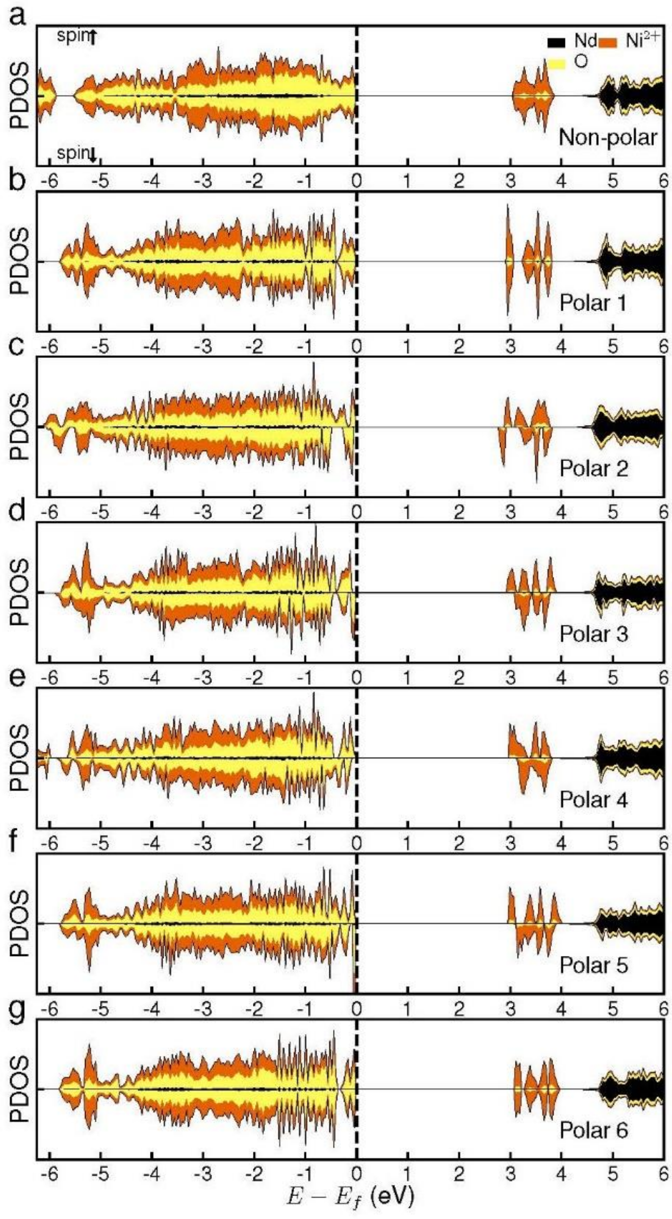

Supplementary Figure S11 | The spin-polarized projected density of states of H-NNO for the non-polar structure as well as the "Polar 1-6" structures.

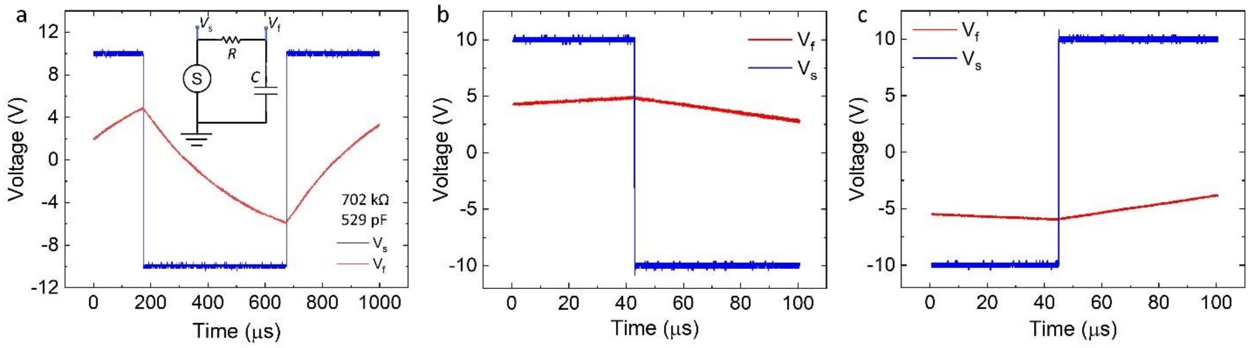

Supplementary Figure S12 | The voltage transients of the applied pulses setup to examine capacitance behavior by replacing the H-NNO capacitor with a commercial multi-layer ceramic capacitor (MLCC-511). (a) The transient response of voltage V_f across the capacitor caused by the square pulse V_S. (b, c) The close-up view of the voltage transient corresponding to (a).

Supplementary Table S3 | Hyperparameters for the FeCAP based neural network simulation.

Parameter	Value
Number of input neurons	784
Number of output neurons	100, 400
Batch size	32
Threshold membrane potential	−52 mV
Resting membrane potential	−65 mV
Reset membrane potential	−65 mV
Refractory period (in terms of timesteps)	5
Number of simulation timesteps	100
Adaptive threshold increment	0.05 mV
Decay time constant of membrane potential (in terms of timesteps)	100
Decay time constant of adaptive threshold (in terms of timesteps)	10^7
Spike trace decay time constant (in terms of timesteps)	20
Weight normalization factor	78.4

Supplementary Note 3 The effect of proton doping concentration on the polarization

To study the effect of the proton doping, we annealed three Pd/NNO/NbSTO samples of 100 nm thickness at different temperatures. The low and moderate doping (SL and SM) were performed at 150 °C and 200 °C separately for 30 minutes under H_2(5%)/Ar(95%) mixture gas. The highest doping (SH) was performed at 250 °C for 8 hours in pure H_2 gas.

Elastic recoil detection analysis (ERDA) was performed to detect the hydrogen concentration in three samples. In Supplementary Figure **S13**a, the spectra show that the hydrogen content increases with the

doping temperature as expected for diffusional process. The averaged H concentrations from SIMNRA modeling of experimental data are given in Supplementary Table **S4**.

The resistance was determined for the three samples by measuring I-V from 0 to 0.1 V and fitting the slope. The resistivity increases with the increase in proton doping concentration (Supplementary Figure **S13**b) and reaches 4.2E+09 $\Omega \cdot \mathrm{cm}$ in the highest doping sample. The resistivity results are consistent with literature[11].

The PUND method was used to measure the remanent polarization for different voltage amplitudes in three different doping samples at room temperature. The PUND loop profiles use the same ramping rate, 32 V/ms, shown in Supplementary Figure **S5**a. Supplementary Figure **S13**c shows the dependence of the extracted remanent polarization on the amplitude of the applied voltage V_{max} in H-NNO samples of different doping levels. At the same voltage, the induced polarization P_{r} decreases with the proton doping level. Higher hydrogenation level results in smaller proton concentration gradient which corresponds to smaller resistance gradient. With the same voltage drop across the thin film, the electric field is smaller and therefore induces less polarization in H-NNO.

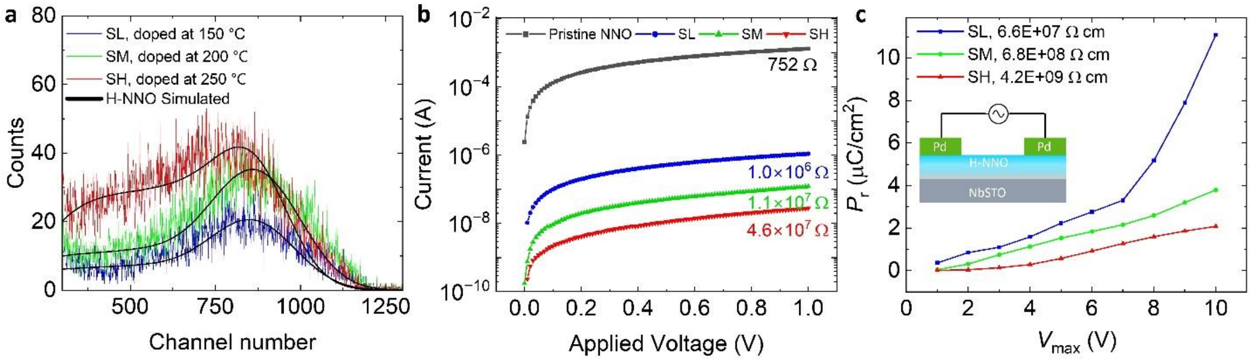

Supplementary Figure S13 | ERDA, transport, and polarization measurements of H-NNO films with different doping levels. (a) ERDA spectra of H-NNO samples doped at 150 °C (SL), 200 °C (SM), and 250 °C (SH) respectively. The energy per channel is 0.67 keV. The thickness of H-NNO is around 100 nm. (b) Room temperature current-voltage (I–V) characteristics for the Pd/NNO/NbSTO device and a series of Pd/H-NNO/NbSTO devices with different doping levels. The resistance which is calculated by fitting from 0 to 0.1 V, increases from 752 to 4.6×10^{7} Ω by proton doping. (c) Remanent polarization measured by the PUND method as a function of the amplitude of the applied voltage for H-NNO samples doped at different temperatures. The highest doping temperature results in the largest resistivity. The device setup is shown in the inset. Due to the conductive NbSTO substrate, the polarization was induced by the out-of-plane electric field.

Supplementary Table S4 | Parameters obtained from ERDA analysis of ~100 nm thick NNO and H-NNO films.

Sample	SL, doped at 150 °C	SM, doped at 200 °C	SH, doped at 250 °C
H/Ni Atomic Ratio	0.1	0.17	0.33

References

1. Sidik, U. *et al.* Tunable Proton Diffusion in NdNiO3 Thin Films under Regulated Lattice Strains. *ACS Appl. Electron. Mater.* **4**, 4849–4856 (2022).

2. Gorbunov, A. V. *et al.* Polarization loss in the organic ferroelectric trialkylbenzene-1,3,5-tricarboxamide (BTA). *Phys. Chem. Chem. Phys.* **19**, 3192–3200 (2017).

3. Jo, J. Y. *et al.* Polarization Switching Dynamics Governed by the Thermodynamic Nucleation Process in Ultrathin Ferroelectric Films. *Phys. Rev. Lett.* **97**, 247602 (2006).

4. Benedetto, J. M., Moore, R. A. & McLean, F. B. Fast decay component of the remanent polarization in thin-film pzt capacitors. *Integr. Ferroelectr.* **1**, 195–204 (1992).

5. Gruverman, A. & Tanaka, M. Polarization retention in SrBi2Ta2O9 thin films investigated at nanoscale. *J. Appl. Phys.* **89**, 1836–1843 (2001).

6. Liu, J., Zhao, Y., Chen, C., Wei, X. & Zhang, Z. Study on the Polarization and Relaxation Processes of Ferroelectric Polymer Films Using the Sawyer-Tower Circuit with Square Voltage Waveform. *J. Phys. Chem. C* **121**, 12531–12539 (2017).

7. Zhu, Y. *et al.* Thickness-Dependent Asymmetric Potential Landscape and Polarization Relaxation in Ferroelectric HfxZr1−xO2 Thin Films through Interfacial Bound Charges. *Adv. Electron. Mater.* **5**, 1900554 (2019).

8. Wang, J. *et al.* Epitaxial BiFeO 3 thin films on Si. *Appl. Phys. Lett.* **85**, 2574–2576 (2004).

9. Yun, K. Y., Ricinschi, D., Kanashima, T., Noda, M. & Okuyama, M. Giant ferroelectric polarization beyond 150 µC/cm2 in BiFeO3 thin film. *Japanese J. Appl. Physics, Part 2 Lett.* **43**, L647 (2004).

10. Wang, Y. *et al.* Chemical-Pressure-Modulated BaTiO3Thin Films with Large Spontaneous Polarization and High Curie Temperature. *J. Am. Chem. Soc.* **143**, 6491–6497 (2021).

11. Sidik, U., Hattori, A. N., Rakshit, R., Ramanathan, S. & Tanaka, H. Catalytic Hydrogen Doping of NdNiO3 Thin Films under Electric Fields. *ACS Appl. Mater. Interfaces* **12**, 54955–54962 (2020).
